# Satisfaction With Telemedicine in Patients With Orthopedic Trauma During the COVID-19 Lockdown: Interview Study

**DOI:** 10.2196/35718

**Published:** 2022-09-12

**Authors:** Thomas Rauer, Julian Scherer, Pascal Stäubli, Jonas Gerber, Hans-Christoph Pape, Sandro-Michael Heining

**Affiliations:** 1 Department of Trauma Surgery University Hospital of Zurich Zurich Switzerland; 2 Faculty of Medicine University of Zurich Zurich Switzerland; 3 Physiotherapy Occupational Therapy University Hospital Zurich Zurich Switzerland

**Keywords:** COVID-19, digital, survey, telehealth, follow-up, orthopedic trauma, trauma, attitude

## Abstract

**Background:**

Telemedicine can take many forms, from telephone-only consultations to video consultations via a smartphone or personal computer, depending on the goals of the treatment. One of the advantages of videoconferencing is the direct visual contact between patients and therapists even over long distances. Although some telemedicine models require specially designed add-on devices, others get by with off-the-shelf equipment and software and achieve similarly successful successful results. This depends, among other things, on the nature of the injury, the desired outcome of therapy, and the medical consultation. In the last decade, the science and practice of telemedicine have grown exponentially and even more so during the COVID-19 pandemic. Depending on the traumatic lesion, posttraumatic and postoperative treatment and care of patients who experience trauma may require medical or physical therapy consultations in a clinic or office. However, due to the COVID-19 lockdown, direct physical follow-up was more difficult, and therefore, telemedicine solutions were sought and implemented.

**Objective:**

The aim of this study was to assess satisfaction with telemedical aftercare in patients with orthopedic trauma.

**Methods:**

Between March and July 2020, a standardized interview using a standardized questionnaire—Freiburg Index of Patient Satisfaction (FIPS)—among patients with orthopedic trauma who received telemedical postsurgical or physiotherapeutic care was conducted. The FIPS is composed of 5 questions regarding treatment and 1 question on the overall treatment satisfaction. Furthermore, we assessed patients’ demographics and their telemedical use. Subgroup analysis was performed for age groups (<65 years vs ≥65 years), the used device, and gender.

**Results:**

In total, we assessed 25 patients with a mean age of 43 (SD 24.31) years (14 female). The majority of patients (n=19, 76%) used their smartphone for consultations. The mean overall FIPS score assessed was 2.14 (SD 0.87). The mean FIPS score for younger patients was 2.23 (SD 0.90) vs 1.91 (SD 0.82) for older patients. The vast majority of the surveyed patients (n=20, 80%) were absolutely confident with their smartphone or tablet use.

**Conclusions:**

Most patients surveyed stated a high satisfaction with the telemedical follow-up. Older patients showed a higher satisfaction rate than their younger counterparts. It seems that telemedical postsurgical or physiotherapeutic care is a viable option, especially in times of reduced contact, like the current COVID-19 pandemic. Thus, telemedicine offers the opportunity to ensure access to effective patient care, even over long distances, while maintaining patient satisfaction.

## Introduction

In the age of digitalization and new communication technologies, almost every generation uses smartphones or computers to be in constant exchange with the environment, to communicate with other people, or to get information anywhere and anytime [1,2]. The term telemedicine is not defined uniformly. Among other definitions, the definition of the European Commission’s Health Care Telematics Program is as follows: “telemedicine is the rapid access to shared and remote medical expertise by means of telecommunication and information technologies, no matter where the patient or relevant information is located” [3]. Accordingly, a key point of telemedical treatment is the rapid exchange of information between the patient and the treating physician. The main reason for this characteristic is the widespread access to technological methods for telemedical communication, further enhanced by the independence of a geographically fixed setting for these consultations. By replacing one-on-one consultations with phone and video calls, patients can eliminate long travels to the clinic and waiting times in the waiting room before visiting the doctor [4]. The resources and costs required for this can be significantly reduced, which makes the interaction between doctor and patient much more cost-efficient [5]. Communication media used for telecommunication are phone calls, emails, videotelephony, SMS, and broadcast or telemedia [6]. Due to the COVID-19 lockdown, many hospitals had to reduce the number of their daily one-on-one consultations [7]. Therefore, they had to find a solution to ensure adequate aftercare of patients [8].

Several previous studies have shown a hypothetical acceptance (if offered by physicians) of postsurgical follow-up or surveillance via telemedical solutions as well as a high willingness to conduct video consultations in general [9,10]. Studies have shown that even physiotherapeutic interventions can be conducted effectively via videoconferencing, which is cost-effective and can give access to patients who live in remote areas [11]. However, a previous study [12] has shown that the COVID-19 crisis had no significant impact on the willingness of patients to use telemedical solutions, and data on patients’ satisfaction with telemedicine, especially during national lockdowns, is lacking. Thus, the aim of this study was to determine the acceptance (subjectively) and satisfaction of telemedicine by patients who have experienced such a telemedical aftercare procedure. A standardized patient satisfaction questionnaire was used to visualize patient satisfaction in a consistent manner. Furthermore, the question of how well patients cope with digital devices, such as smartphones, computers, and tablets, was answered.

## Methods

### Patients and Setting

During the COVID-19 lockdown from March to July 2020, the department of a European Level 1 trauma center offered selected patients, for whom one-on-one therapy was not medically imperative, the option of telemedicine follow-up or physical therapy in lieu of a one-on-one consultation. Patients included in this study consented verbally to telemedical care and had the technical requirements (ie, smartphone, computer, or phone) and expertise (subjectively) needed to attend the telemedical consultation. The telemedical follow-up or physical therapy took place either as a pure telephone consultation or as a videotelephone consultation by means of a smartphone or computer with integrated video function, where the patient could demonstrate findings such as wounds and range of motion to the treating therapist, or the therapist could instruct the patient on appropriate physiotherapeutic exercises.

Patients who medically required a one-on-one consultation, those who were declined telemedical follow-up or treatment, or could not meet the technical requirements for other reasons, such as not owning a smartphone, tablet, or computer, or not having an internet connection, were excluded.

Patients who received a telemedical follow-up or treatment were called by phone and asked to retrospectively complete a standardized patient satisfaction questionnaire (Freiburg Index of Patient Satisfaction) in relation to the treatment that had taken place after the postoperative treatment was ended. In addition, they were asked what type of device they used for telemedicine consultations and whether they are familiar with using smartphones and tablets. The questionnaire and the additional question were then completed during the telephone call as part of a standardized interview.

### Freiburg Index of Patient Satisfaction

The standardized questionnaire used in this study was the Freiburg Index of Patient Satisfaction (FIPS) questionnaire, which was developed in 2013 by Miernik et al [13] to assess treatment-related patient satisfaction. The questionnaire can be used across disciplines and regardless of the type of interventional or operative treatment. The questionnaire consists of 5 questions, called items, which are assessed using a 6-point scheme. Questions 1 to 4 can be rated on a scale from 1 (strongly agree) to 6 (strongly disagree). Question 5 can be rated on a scale from 1 (excellent) to 6 (very poor). The sum value of the points awarded is divided by the number of items after all questions have been answered, resulting in an overall score, the FIPS score. The FIPS score is thus between 1 and 6, with 1 corresponding to an excellent score and 6 to a very poor score. With the help of a regression analysis, Miernik et al [13] were able to show that neither the invasiveness of the procedure nor sociodemographic factors, such as age, gender, or school leaving certificate, have any influence on patient satisfaction. The independence of sociodemographic factors is very unusual, as they have been shown to be influencing factors in many studies. This underlines the ubiquitous applicability of the FIPS questionnaire [13-15].

The questionnaire is formulated in a way that is very understandable for everyone and can therefore be answered completely by almost all patients independently. The FIPS questionnaire is considered valid, reliable, and one-dimensional, meaning that it only focuses on subjective patient satisfaction [13]. However, patient satisfaction alone cannot provide a final verdict on the quality of a treatment or therapy, as it provides a subjective picture. Nevertheless, it is an important parameter for establishing a comprehensive quality assessment of a treatment. For the optimal validity, patient satisfaction as well as clinical parameters or scores should be included in the overall assessment.

### Statistical Analysis

Further statistical analysis was performed with the use of IBM SPSS Statistics for Mac (version 26.0; IBM Corp). Data are presented as frequencies (n) and means with SDs. To assess differences in ordinal data between the groups, a nonparametric median test and a chi-square test for nominal data were used. A subgroup analysis was performed for the age groups (<65 years vs ≥65 years), gender, and the used device. The level of statistical significance was set at *P*<.05.

### Ethical Considerations

The local ethics committee (Kantonale Ethikkommission, Kanton Zürich) ruled that no ethical approval was necessary for this study (BASEC-Nr. Req-2020-00562).

## Results

### Demographics

In total, 25 patients (14 female) with a mean age of 43 (SD 24.31; range 14-95) years were included. Of them, 7 patients were 65 years or older. Most patients (n=19, 76%) used a smartphone for the telemedical consultations, 4 (16%) used a computer, and 2 (8%) used a landline phone. There was no difference between female and male patients (*P*=.42) and between age groups (*P*=.06).

### FIPS Questionnaire

**Figure 1:**
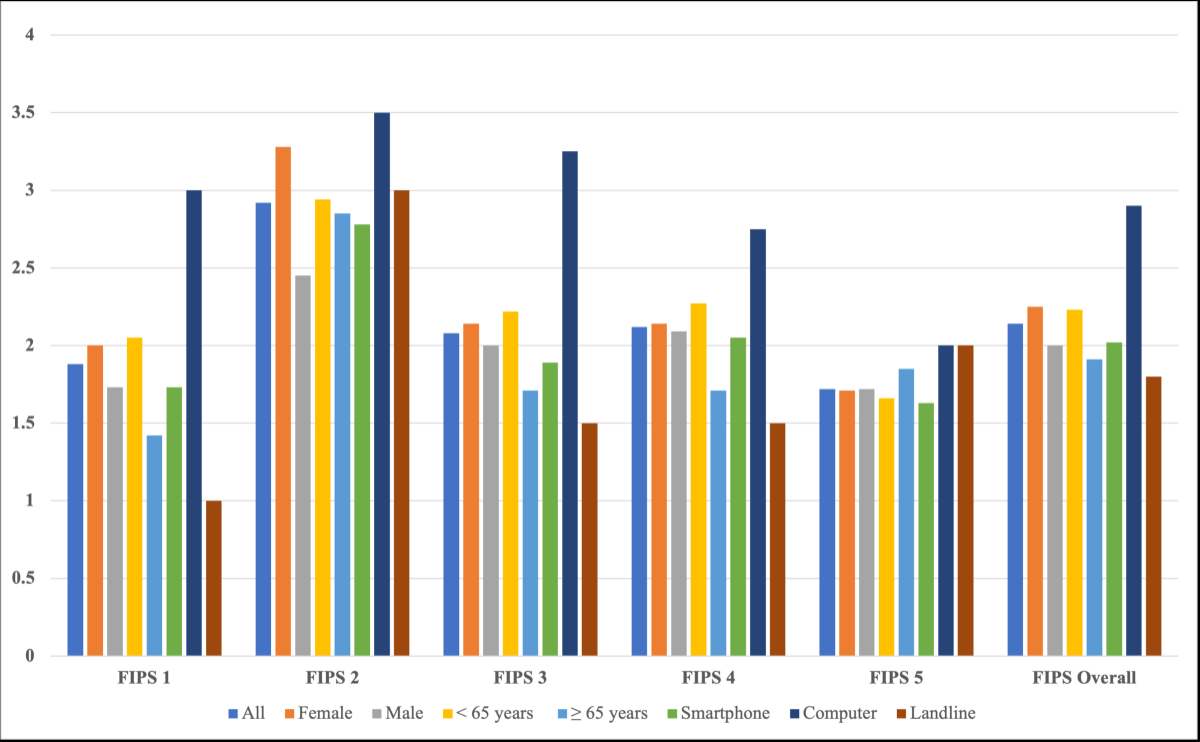
Freiburg Index of Patient Satisfaction (FIPS) ratings stratified by subgroups.

The results of the FIPS Questionnaire can be found in Figure 1 and Table S1 in Multimedia Appendix 1.

#### 1. Burden of Treatment

The mean overall score for this question was 1.88 (SD 1.23; range 1-5). There was no difference between female and male patients (*P*=.56). Patients over 65 years (n=7, 28%) rated this question significantly better than their younger counterparts (*P*=.04). Patients using a smartphone and landline phone had significantly better ratings than patients using a computer (*P*=.04).

#### 2. Recovery After Treatment

The mean overall score for this question was 2.92 (SD 1.55; range 1-6). Male patients rated this question insignificantly better than their female counterparts (*P*=.07). There was no difference between the assessed age groups (*P*=.57). No difference was seen between the used devices (*P*=.12).

#### 3. Success of the Treatment

The mean overall score for this question was 2.08 (SD 1.04; range 1-5). No differences were seen in regard to gender (*P*=.84) as well as between the age groups (*P*=.66). Smartphone users and landline phone users showed a significantly better rating than those who used a computer (*P*=.02).

#### 4. Willingness to Repeat the Treatment

The mean overall score for this question was 2.12 (SD 1.30; range 1-5). No differences were seen in regard to gender (*P*=.55) as well as between the age groups (*P*=.48). No difference was seen between the used devices (*P*=.71).

#### 5. Overall Rating of the Treatment

The mean score among all participants for the overall rating was 1.72 (SD 0.79; range 1-4). No differences were seen in regard to gender (*P*=.40) as well as between the age groups (*P*=.83). No difference was seen between the used devices (*P*=.62).

#### 6. Overall FIPS

The mean overall FIPS score among all patients was 2.14 (SD 0.87; range 1-4). The mean FIPS score for younger patients was 2.23 (SD 0.90) vs 1.91 (SD 0.82) for older patients. There was no significant difference between female and male patients (*P*=.69). There was also no difference between younger and older patients (*P*=.33). There was no statistical difference between the devices used (*P*=.21).

### Familiarity With Smartphones and Computers or Tablets

**Figure 2:**
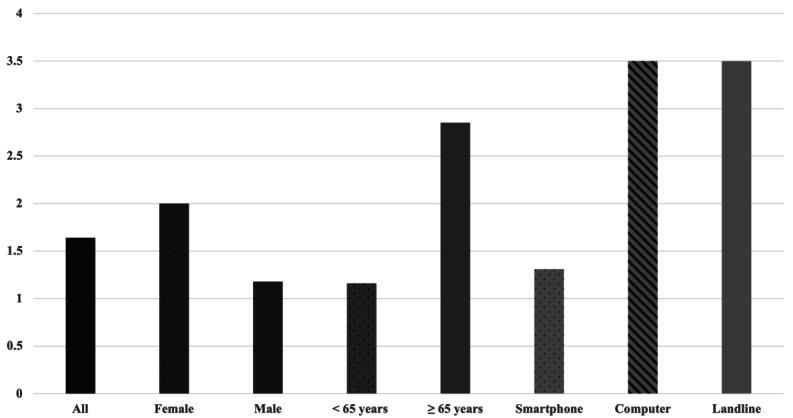
Familiarity with smartphones and computers or tablets.

Additionally, we found a mean score of 1.64 (SD 1.49; range 1-6) regarding electronic device familiarity among all patients. The vast majority of the surveyed patients (n=20, 80%) were absolutely confident with their smartphone or tablet use. There was no significant difference between female and male patients (*P*=.23). There was also no difference between younger and older patients (*P*=.08). Regarding device use, there was no significant difference between the groups (*P*=.11; Figure 2).

## Discussion

### Principal Findings and Comparison With Prior Work

The aim of this study was to investigate satisfaction with telemedical consultations in patients with orthopedic trauma during the COVID-19 pandemic. The answers of the 25 included patients to the FIPS questionnaire showed very positive results, reflected by an average FIPS score of 2.14 (SD 0.87). Interestingly, patients older than 65 years of age scored in the “burden of treatment” question significantly better than their younger counterparts. Furthermore, they scored the treatment in almost every item of the FIPS better than patients younger than 65 years of age. This is interesting, since other studies suggest that older patients are rather less interested in telemedicine than younger patients [5,9]. Interestingly, patients aged more than 65 years stated that they were less familiar with their electronic devices than younger patients but rated a better score for the actual telemedical treatment. The authors suggest that this indicates the easy use of telemedical devices after treatment, showing that it does not require advanced technical skills. This is all the more true for smartphones, which have become widely used in all social classes and age groups in recent years. This could be one explanation why smartphone users and also landline phone users rated the success of the therapy significantly better than their counterparts who used a computer. No significant differences between male and female patients were found in our study. Male patients showed slightly higher confidence with their electronic devices and showed a slightly better FIPS rating than female participants. This is contrary to several studies, which showed higher use of and satisfaction with telemedicine in female patients [12]. However, a recent study on telemedical use during the COVID-19 pandemic showed findings similar to our study. Ramaswamy et al [16] assessed that younger age and female gender were associated with lower satisfaction after telemedical treatment. However, they were also able to show that during the COVID-19 pandemic, patients treated with telemedical solutions showed higher satisfaction compared to one-on-one treatments. This result supports the idea of introducing telemedical consultations with videotelephony as an alternative to one-on-one consultations. These findings as well as the results of our study suggest that telemedical solutions are valid options in terms of cost-effectiveness and travel times and especially in times of a pandemic when personal contact avoidance is desired. To our knowledge, no such findings have been described in the current literature.

### Limitations

This study has certain limitations. It is well known that surveys have minor levels of evidence, and the outcome of this study is directly connected to participant’s understanding of the questionnaire. Although there are clear trends seen in our results, these findings should be treated with caution, considering the broad age spectrum and the very diverse patient population. Patients selected for a telemedical consultation were required to have the necessary prerequisites, such as internet connection, a telemedicine-enabled digital device, and the expertise needed to operate it. This causes a certain bias in the results obtained, as patients were already selected and were sympathetic toward telemedical consultations.

As another limitation, it may be noted that we did not focus on patients with a specific injury and included patients with different patterns of injury. However, because the focus of this study was on overall patient satisfaction with telemedical treatment regardless of injury pattern, we consider the inclusion of patients with different injury patterns to have a negligible bias on the significance regarding patient satisfaction.

In addition, the limited number of included patients limits the generalizability of the statements. However, the statistically significant findings can be considered highly significant with such a small cohort.

### Conclusions

The majority of patients surveyed stated a high satisfaction with the telemedical follow-up. The results of this survey showed a positive trend in patients’ attitudes toward telemedicine in both age groups, with a higher satisfaction rate in the group of older patients.

It seems that telemedical postsurgical or physiotherapeutic care is a viable option, especially in times of reduced contact, like the current COVID-19 pandemic. Our study is another component to fill the gap in the available literature on telemedicine in the treatment of patients who experience orthopedic trauma.

Further studies should include a larger number of patients and focus specifically on different trauma entities. Furthermore, a matched-pair analysis, assessing differences between telemedical aftercare and conventional aftercare, should be performed.

## Appendix

Multimedia Appendix 1

## Abbreviations

FIPS: Freiburg Index of Patient Satisfaction
