# Supplementary material for: Satisfaction With Telemedicine in Patients With Orthopedic Trauma During the COVID-19 Lockdown: Interview Study
Source: JMIR Form Res. 2022 Sep 12;6(9):e35718. doi: 10.2196/35718 (PMC9472502; doi:10.2196/35718)
Supplement: Multimedia Appendix 1 [file formative_v6i9e35718_app1.docx]

|  | All | Female | Male | < 65 years | ≥ 65 years | Smartphone | Computer | Landline |
| --- | --- | --- | --- | --- | --- | --- | --- | --- |
| *Burden of treatment* | 1.88 | 2.00 | 1.73 | 2.05 | 1.42* | 1.73* | 3.00 | 1.00* |
| *Recovery after treatment* | 2.92 | 3.28 | 2.45 | 2.94 | 2.85 | 2.78 | 3.50 | 3.00 |
| *Success of the treatment* | 2.08 | 2.14 | 2.00 | 2.22 | 1.71 | 1.89* | 3.25 | 1.50* |
| *Repetition of the treatment* | 2.12 | 2.14 | 2.09 | 2.27 | 1.71 | 2.05 | 2.75 | 1.50 |
| *Overall rating of the treatment* | 1.72 | 1.71 | 1.72 | 1.66 | 1.85 | 1.63 | 2.00 | 2.00 |
| *Overall FIPS* | 2.14 | 2.25 | 2.00 | 2.23 | 1.91 | 2.02 | 2.90 | 1.80 |
